# 3-(2-Fluoro­phen­yl)-1-(4-methoxy­phen­yl)prop-2-en-1-one

**DOI:** 10.1107/S1600536809045759

**Published:** 2009-11-07

**Authors:** Huan-Mei Guo

**Affiliations:** a Microscale Science Institute, Weifang University, Weifang 261061, People’s Republic of China

## Abstract

The title compound, C_16_H_13_FO_2_, was prepared from 4-methoxy­hypnone and 2-fluoro­benzophenone by a Claisen–Schmidt condensation reaction. The dihedral angle between the two benzene rings is 31.99 (2)°. In the crystal structure, mol­ecules are linked by weak inter­molecular C—H⋯O hydrogen bonds along [010].

## Related literature

For the biological activity of chalcones, see: Hsieh *et al.* (1998 ▶); Anto *et al.* (1994 ▶); De Vincenzo *et al.* (2000 ▶); Dimmock *et al.* (1998 ▶). For related structures, see: Fun *et al.* (2008 ▶); Zhao *et al.* (2009 ▶).
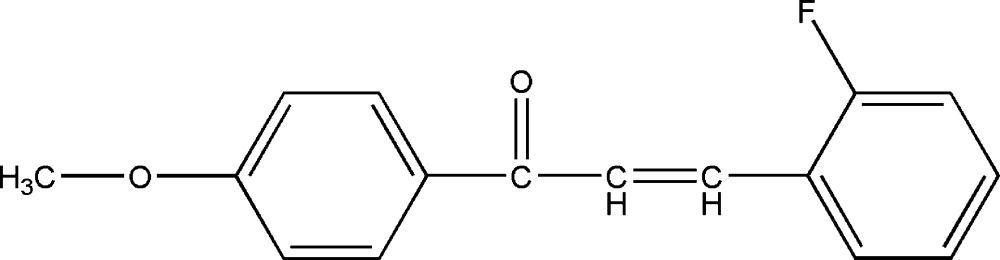


## Experimental

### 

#### Crystal data

C_16_H_13_FO_2_
*M* *_r_* = 256.26
Orthorhombic, 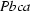

*a* = 7.4511 (6) Å
*b* = 11.0541 (8) Å
*c* = 31.031 (3) Å
*V* = 2555.9 (3) Å^3^
*Z* = 8
Mo *K*α radiation
μ = 0.10 mm^−1^
*T* = 298 K
0.30 × 0.20 × 0.15 mm

#### Data collection

Bruker SMART CCD diffractometer
Absorption correction: none
15509 measured reflections
3162 independent reflections
2162 reflections with *I* > 2σ(*I*)
*R*_int_ = 0.026

#### Refinement

*R*[*F* ^2^ > 2σ(*F* ^2^)] = 0.042
*wR*(*F* ^2^) = 0.117
*S* = 1.05
3162 reflections
173 parameters
H-atom parameters constrained
Δρ_max_ = 0.16 e Å^−3^
Δρ_min_ = −0.19 e Å^−3^

### 

Data collection: *SMART* (Bruker, 1997 ▶); cell refinement: *SAINT* (Bruker, 1997 ▶); data reduction: *SAINT*; program(s) used to solve structure: *SHELXS97* (Sheldrick, 2008 ▶); program(s) used to refine structure: *SHELXL97* (Sheldrick, 2008 ▶); molecular graphics: *SHELXTL* (Sheldrick, 2008 ▶); software used to prepare material for publication: *SHELXTL*.

## Supplementary Material

Crystal structure: contains datablocks global, I. DOI: 10.1107/S1600536809045759/lh2941sup1.cif

Structure factors: contains datablocks I. DOI: 10.1107/S1600536809045759/lh2941Isup2.hkl

Additional supplementary materials:  crystallographic information; 3D view; checkCIF report

## Figures and Tables

**Table 1:** Hydrogen-bond geometry (Å, °)

*D*—H⋯*A*	*D*—H	H⋯*A*	*D*⋯*A*	*D*—H⋯*A*
C11—H11*A*⋯O2^i^	0.93	2.51	3.3679 (18)	153

Symmetry code: (i) 
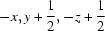
.

**Fig. 1.**
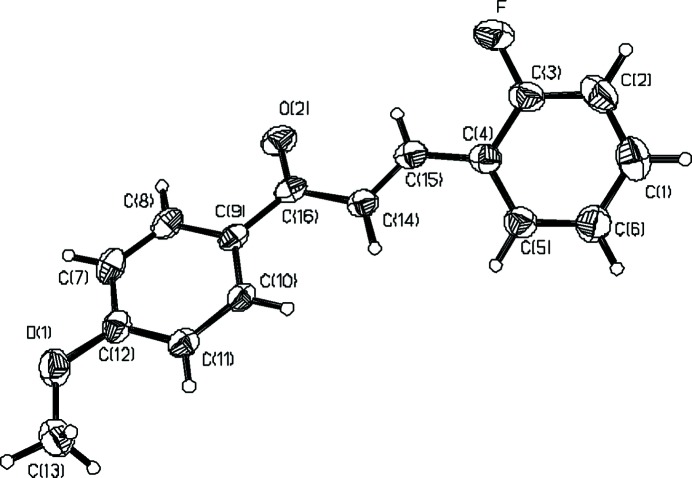
The molecular structure of the title compound with the atom-labeling scheme. Displacement ellipsoids are drawn at the 30% probability level.

## supplementary crystallographic information

### Comment

Chalcones have been identified as interesting compounds having multiple biological activities which include antiinflammatory (Hsieh *et al.*,1998) and antioxidant (Anto *et al.*,1994). The effectiveness of chalcone compounds against cancer has been investigated (De Vincenzo *et al.*,2000;Dimmock *et al.*,1998). As part of our search for new biologically active compounds we synthesized the title compound (I) and report its crystal structure herein.

The molecular structure of (I) is shown in Fig.1. The dihedral angle between the two benzene rings (C1—C6 and C7—C12) is 31.99 (2)°. The bond lengths and bond angles are within normal ranges and comparable to those in a related structures (Fun *et al.*, 2008; Zhao *et al.*, 2009). In the crystal structure, molecules are linked by weak intermolecular C-H···O hydrogen bonds into one-dimensional chains along [010] (Fig. 2).

### Experimental

A mixture of 4-methoxyhypnone (0.02 mol) and 2-fluorobenzophenone (0.02 mol) and 10% NaOH (10ml) was stirred in ethanol (30 ml) for 2 h to afford the title compound (yield 85%). Single crystals suitable for X-ray measurements were obtailed by recrystallization of an ethyl acetate solution of the title compound at room temperature.

### Refinement

H atoms were positioned geometrically and allowed to ride on their parent atoms, with C—H distances of 0.93–0.96 Å, and with *U*_iso_(H) = 1.2*U*_eq_ of the parent atoms.

### Crystal data

**Table:**

C_16_H_13_FO_2_	*F*(000) = 1072
*M**_r_* = 256.26	*D*_x_ = 1.332 Mg m^−^^3^
Orthorhombic, *P**b**c**a*	Mo *K*α radiation, λ = 0.71073 Å
Hall symbol: -P 2ac 2ab	Cell parameters from 2162 reflections
*a* = 7.4511 (6) Å	θ = 2.6–28.4°
*b* = 11.0541 (8) Å	µ = 0.10 mm^−^^1^
*c* = 31.031 (3) Å	*T* = 298 K
*V* = 2555.9 (3) Å^3^	Bar, yellow
*Z* = 8	0.30 × 0.20 × 0.15 mm

### Data collection

**Table:**

Bruker SMART CCD diffractometer	2162 reflections with *I* > 2σ(*I*)
Radiation source: fine-focus sealed tube	*R*_int_ = 0.026
graphite	θ_max_ = 28.4°, θ_min_ = 2.6°
φ and ω scans	*h* = −8→9
15509 measured reflections	*k* = −13→14
3162 independent reflections	*l* = −39→36

### Refinement

**Table:**

Refinement on *F*^2^	Secondary atom site location: difference Fourier map
Least-squares matrix: full	Hydrogen site location: inferred from neighbouring sites
*R*[*F*^2^ > 2σ(*F*^2^)] = 0.042	H-atom parameters constrained
*wR*(*F*^2^) = 0.117	*w* = 1/[σ^2^(*F*_o_^2^) + (0.0479*P*)^2^ + 0.3713*P*] where *P* = (*F*_o_^2^ + 2*F*_c_^2^)/3
*S* = 1.05	(Δ/σ)_max_ < 0.001
3162 reflections	Δρ_max_ = 0.16 e Å^−^^3^
173 parameters	Δρ_min_ = −0.19 e Å^−^^3^
0 restraints	Extinction correction: *SHELXL97* (Sheldrick, 2008), Fc^*^=kFc[1+0.001xFc^2^λ^3^/sin(2θ)]^-1/4^
Primary atom site location: structure-invariant direct methods	Extinction coefficient: 0.0083 (9)

### Special details

**Table:**

Geometry. All e.s.d.'s (except the e.s.d. in the dihedral angle between two l.s. planes) are estimated using the full covariance matrix. The cell e.s.d.'s are taken into account individually in the estimation of e.s.d.'s in distances, angles and torsion angles; correlations between e.s.d.'s in cell parameters are only used when they are defined by crystal symmetry. An approximate (isotropic) treatment of cell e.s.d.'s is used for estimating e.s.d.'s involving l.s. planes.
Refinement. Refinement of *F*^2^ against ALL reflections. The weighted *R*-factor *wR* and goodness of fit *S* are based on *F*^2^, conventional *R*-factors *R* are based on *F*, with *F* set to zero for negative *F*^2^. The threshold expression of *F*^2^ > σ(*F*^2^) is used only for calculating *R*-factors(gt) *etc*. and is not relevant to the choice of reflections for refinement. *R*-factors based on *F*^2^ are statistically about twice as large as those based on *F*, and *R*- factors based on ALL data will be even larger.

### Fractional atomic coordinates and isotropic or equivalent isotropic displacement parameters (Å^2^)

**Table:**

	*x*	*y*	*z*	*U*_iso_*/*U*_eq_	
C9	0.14251 (16)	0.07378 (11)	0.24089 (4)	0.0474 (3)	
C16	0.16025 (17)	0.04544 (11)	0.19449 (5)	0.0530 (3)	
O2	0.20708 (16)	−0.05586 (9)	0.18272 (3)	0.0723 (3)	
C11	0.06705 (19)	0.20676 (12)	0.30007 (4)	0.0539 (3)	
H11A	0.0271	0.2817	0.3097	0.065*	
C10	0.08333 (18)	0.18431 (11)	0.25647 (4)	0.0507 (3)	
H10A	0.0537	0.2451	0.2370	0.061*	
C12	0.11063 (18)	0.11693 (12)	0.32916 (4)	0.0548 (3)	
C15	0.0866 (2)	0.11137 (13)	0.12171 (5)	0.0598 (4)	
H15A	0.1029	0.0307	0.1142	0.072*	
C14	0.11574 (19)	0.13923 (12)	0.16225 (5)	0.0565 (4)	
H14A	0.1082	0.2197	0.1709	0.068*	
O1	0.09938 (16)	0.12870 (10)	0.37267 (3)	0.0747 (3)	
C4	0.03134 (19)	0.19281 (13)	0.08723 (4)	0.0567 (4)	
C8	0.1842 (2)	−0.01562 (12)	0.27107 (5)	0.0597 (4)	
H8A	0.2232	−0.0909	0.2616	0.072*	
F	−0.04947 (18)	0.02464 (10)	0.04543 (3)	0.1056 (4)	
C7	0.1688 (2)	0.00549 (13)	0.31425 (5)	0.0656 (4)	
H7A	0.1975	−0.0553	0.3338	0.079*	
C5	0.0374 (2)	0.31898 (13)	0.09031 (5)	0.0638 (4)	
H5A	0.0862	0.3546	0.1148	0.077*	
C3	−0.0402 (2)	0.14689 (15)	0.04940 (5)	0.0698 (4)	
C6	−0.0271 (3)	0.39113 (16)	0.05794 (5)	0.0783 (5)	
H6A	−0.0227	0.4748	0.0608	0.094*	
C13	0.0495 (2)	0.24277 (16)	0.39010 (5)	0.0743 (5)	
H13A	0.0465	0.2376	0.4210	0.112*	
H13B	−0.0671	0.2649	0.3796	0.112*	
H13C	0.1354	0.3028	0.3815	0.112*	
C2	−0.1052 (3)	0.21693 (19)	0.01653 (5)	0.0822 (5)	
H2A	−0.1525	0.1818	−0.0082	0.099*	
C1	−0.0987 (3)	0.3404 (2)	0.02105 (5)	0.0849 (5)	
H1A	−0.1426	0.3900	−0.0008	0.102*	

### Atomic displacement parameters (Å^2^)

**Table:**

	*U*^11^	*U*^22^	*U*^33^	*U*^12^	*U*^13^	*U*^23^
C9	0.0380 (6)	0.0385 (6)	0.0657 (8)	−0.0049 (5)	−0.0016 (5)	0.0041 (5)
C16	0.0447 (7)	0.0411 (7)	0.0731 (9)	−0.0071 (5)	0.0028 (6)	−0.0035 (6)
O2	0.0834 (8)	0.0458 (6)	0.0876 (8)	0.0051 (5)	0.0089 (6)	−0.0080 (5)
C11	0.0565 (8)	0.0436 (7)	0.0616 (8)	−0.0001 (6)	−0.0029 (6)	0.0026 (6)
C10	0.0531 (8)	0.0393 (6)	0.0597 (8)	−0.0011 (6)	−0.0050 (6)	0.0066 (5)
C12	0.0481 (7)	0.0579 (8)	0.0585 (8)	−0.0039 (6)	−0.0017 (6)	0.0110 (6)
C15	0.0627 (9)	0.0495 (7)	0.0672 (9)	−0.0063 (7)	0.0071 (7)	−0.0087 (6)
C14	0.0617 (9)	0.0461 (7)	0.0616 (8)	−0.0032 (6)	0.0036 (6)	−0.0055 (6)
O1	0.0864 (8)	0.0769 (7)	0.0608 (7)	0.0062 (6)	−0.0007 (5)	0.0141 (5)
C4	0.0545 (8)	0.0621 (8)	0.0535 (8)	−0.0062 (7)	0.0087 (6)	−0.0085 (6)
C8	0.0562 (8)	0.0419 (7)	0.0809 (10)	0.0065 (6)	0.0026 (7)	0.0073 (6)
F	0.1581 (12)	0.0820 (7)	0.0768 (7)	−0.0307 (7)	−0.0027 (6)	−0.0256 (5)
C7	0.0654 (9)	0.0541 (8)	0.0773 (10)	0.0088 (7)	−0.0005 (8)	0.0223 (7)
C5	0.0702 (10)	0.0624 (9)	0.0587 (8)	−0.0028 (8)	0.0039 (7)	−0.0065 (7)
C3	0.0782 (11)	0.0721 (10)	0.0592 (9)	−0.0144 (8)	0.0106 (8)	−0.0159 (8)
C6	0.0958 (13)	0.0692 (10)	0.0698 (10)	0.0077 (9)	0.0080 (9)	0.0008 (8)
C13	0.0755 (11)	0.0871 (12)	0.0604 (9)	−0.0049 (9)	−0.0012 (8)	0.0005 (8)
C2	0.0839 (12)	0.1103 (15)	0.0525 (9)	−0.0108 (11)	0.0016 (8)	−0.0104 (9)
C1	0.0877 (13)	0.1064 (15)	0.0607 (10)	0.0128 (11)	0.0051 (9)	0.0075 (10)

### Geometric parameters (Å, °)

**Table:**

C9—C10	1.3859 (18)	C4—C5	1.399 (2)
C9—C8	1.3964 (18)	C8—C7	1.365 (2)
C9—C16	1.4794 (19)	C8—H8A	0.9300
C16—O2	1.2285 (15)	F—C3	1.3587 (19)
C16—C14	1.478 (2)	C7—H7A	0.9300
C11—C12	1.3807 (18)	C5—C6	1.370 (2)
C11—C10	1.3808 (19)	C5—H5A	0.9300
C11—H11A	0.9300	C3—C2	1.369 (2)
C10—H10A	0.9300	C6—C1	1.382 (2)
C12—O1	1.3591 (17)	C6—H6A	0.9300
C12—C7	1.385 (2)	C13—H13A	0.9600
C15—C14	1.3134 (19)	C13—H13B	0.9600
C15—C4	1.457 (2)	C13—H13C	0.9600
C15—H15A	0.9300	C2—C1	1.373 (3)
C14—H14A	0.9300	C2—H2A	0.9300
O1—C13	1.421 (2)	C1—H1A	0.9300
C4—C3	1.386 (2)		
			
C10—C9—C8	117.44 (13)	C7—C8—H8A	119.4
C10—C9—C16	123.68 (12)	C9—C8—H8A	119.4
C8—C9—C16	118.87 (12)	C8—C7—C12	120.40 (13)
O2—C16—C14	120.11 (13)	C8—C7—H7A	119.8
O2—C16—C9	120.52 (13)	C12—C7—H7A	119.8
C14—C16—C9	119.34 (11)	C6—C5—C4	121.29 (15)
C12—C11—C10	119.38 (13)	C6—C5—H5A	119.4
C12—C11—H11A	120.3	C4—C5—H5A	119.4
C10—C11—H11A	120.3	F—C3—C2	118.48 (15)
C11—C10—C9	121.88 (12)	F—C3—C4	117.44 (15)
C11—C10—H10A	119.1	C2—C3—C4	124.06 (16)
C9—C10—H10A	119.1	C5—C6—C1	120.44 (17)
O1—C12—C11	124.49 (13)	C5—C6—H6A	119.8
O1—C12—C7	115.86 (12)	C1—C6—H6A	119.8
C11—C12—C7	119.65 (13)	O1—C13—H13A	109.5
C14—C15—C4	127.23 (13)	O1—C13—H13B	109.5
C14—C15—H15A	116.4	H13A—C13—H13B	109.5
C4—C15—H15A	116.4	O1—C13—H13C	109.5
C15—C14—C16	121.39 (13)	H13A—C13—H13C	109.5
C15—C14—H14A	119.3	H13B—C13—H13C	109.5
C16—C14—H14A	119.3	C3—C2—C1	118.28 (16)
C12—O1—C13	118.62 (12)	C3—C2—H2A	120.9
C3—C4—C5	115.82 (14)	C1—C2—H2A	120.9
C3—C4—C15	120.28 (14)	C2—C1—C6	120.11 (17)
C5—C4—C15	123.83 (13)	C2—C1—H1A	119.9
C7—C8—C9	121.24 (13)	C6—C1—H1A	119.9

### Hydrogen-bond geometry (Å, °)

**Table:**

*D*—H···*A*	*D*—H	H···*A*	*D*···*A*	*D*—H···*A*
C11—H11A···O2^i^	0.93	2.51	3.3679 (18)	153

Symmetry codes: (i) −*x*, *y*+1/2, −*z*+1/2.
